# Are Kidney-Tonifying and Blood-Activating Medicinal Herbs Better than NSAIDs for Knee Osteoarthritis? A Systematic Review and Meta-Analysis

**DOI:** 10.1155/2019/9094515

**Published:** 2019-11-25

**Authors:** Hetao Huang, Jianke Pan, Weiyi Yang, Yanhong Han, Minghui Luo, Haodong Liang, Lingfeng Zeng, Guihong Liang, Jiongtong Lin, Jun Liu

**Affiliations:** ^1^Second School of Clinical Medicine, Guangzhou University of Chinese Medicine, Guangzhou 510405, China; ^2^Department of Orthopaedics, Second Affiliated Hospital of Guangzhou University of Chinese Medicine, Guangdong Provincial Hospital of Chinese Medicine, Guangzhou 510120, China; ^3^Guangzhou Hospital of Traditional Chinese Medicine, Guangzhou 510130, China

## Abstract

**Objective:**

To compare the efficacy and safety of kidney-tonifying and blood-activating medicinal herbs (KTBAMs) and nonsteroidal anti-inflammatory drugs (NSAIDs) in the treatment of knee osteoarthritis (KOA).

**Methods:**

Randomized controlled trials (RCTs) from online databases that compared the efficacy of KTBAMs and NSAIDs in the treatment of KOA were retrieved. The main outcomes included the evaluation of functional outcomes, pain, and adverse effects. The Cochrane risk-of-bias (ROB) tool was used to assess methodological quality.

**Results:**

A total of 38 RCTs (3994 participants) were included in our meta-analysis. We found that KTBAMs had a significantly higher total effective rate (*P* < 0.00001, risk ratio (RR) = 1.08, confidence interval (CI) = 1.05 to 1.11, *I*^2^ = 4%) and a lower gastrointestinal adverse reaction rate (*P* < 0.00001, RR = 0.36, CI = 0.24 to 0.53, *I*^2^ = 33%) than NSAIDs. KTBAMs showed greater improvements in the Knee Society Scale (KSS) scores (mean difference (MD) = 7.17, 95% CI 0.71 to 13.64, *P*=0.03). Regarding the visual analog scale (VAS) scores, WOMAC scores, and Lequence scores, there were no significant differences between the KTBAM group and the NSAID group. The GRADE quality level of this systematic review indicated that the very low-quality evidence showed that KTBAMs had a higher total effective rate, while the moderate-quality evidence showed that the adverse reactions of KTBAMs were lower and the KSS scores were higher. Low-quality evidence showed no significant differences in improving VAS scores, WOMAC scores, or Lequence scores.

**Conclusion:**

KTBAMs were superior to NSAIDs in terms of a higher total effective rate, a lower adverse reaction rate, and a higher KSS score. There were no significant differences between KTBAMs and NSAIDs in improving VAS scores, WOMAC scores, and Lequence scores of patients with KOA. Therefore, KTBAMs may be an alternative effective method for treating KOA. However, high-quality, well-designed RCTs with long-term follow-up are still required.

## 1. Introduction

Knee osteoarthritis (KOA) is one of the most common chronic muscular diseases in old people [1]. The main manifestations of KOA are pain and dysfunction in the knees, which affect quality of life and lead to a high rate of disability in elderly individuals. The approximate prevalence of KOA in the general population throughout the world is 12%–35% [2]. KOA has a heavy socioeconomic burden in developed countries. Recently, KOA has become one of the global burden diseases. In some Asian countries, the high prevalence of KOA has increased medical care expenditures and attracted much government attention [3].

The main objectives in the management of KOA have been to alleviate pain, educate patients about their disease, restore function, slow down the progression of disease, and maintain a health-related quality of life [4]. Traditionally, the management of end-stage KOA for relieving pain and improving function has been knee arthroplasty [1]. Conservative approaches address early stages of the disease, such as oral NSAIDs, hyaluronic injection, and self-management, but the clinical results may not satisfy patients. In light of this situation, alternative treatments such as herbal preparations [5], acupuncture [6], moxibustion [7], massage [8], and tai-chi [9] have been investigated for their efficacy in randomized controlled trials (RCTs) and have drawn attention.

As an alternative therapy, Chinese herbal medicine (CHM) or herbal products have been used and recommended by many clinicians. These have been indicated to help alleviate KOA symptoms and reduce costs [10–13]. KTBAMs are one type of Chinese herbal recipe consisting of herbals that can “tonify kidney” and “activate blood” based on traditional Chinese theory. Research on the mechanism of some recipes of KTBAMs have shown their effectiveness in promoting chondrocyte proliferation, inhibiting sodium nitroprussiate-induced chondrocyte apoptosis, and regulating the expression of vascular endothelial growth factor (VEGF) and hypoxia-inducible factor-1*α* (HIF-1*α*) [14–18]. Recently, researchers have reported that KTBAMs can help control KOA-related symptoms and have been widely used in many Asian countries [19]. NSAIDs are the most popular medicine because of their promising effect for KOA, although they are accompanied by high costs and many related side effects [20]. KTBAMs, alone or combined with conventional pharmaceutical drugs, have also been commonly used for the clinical management of KOA. Some researchers [21, 22] have published systematic reviews of the efficacy and safety of traditional Chinese medicine prescriptions in the treatment of KOA. However, most of the systematic reviews have been based on intervention measures that include “traditional Chinese medicine,” but there is no systematic review of a specific kind of traditional Chinese medicine. In particular, no study has systematically examined the effectiveness and safety of KTBAMs for KOA according to the Preferred Reporting Items for Systematic reviews and Meta-Analyses (PRISMA) until now. To assist clinical practice and possibly to reduce the heavy burden of KOA patients, it is important to systematically review the current evidence of KTBAMs compared with NSAIDS. Thus, we performed a meta-analysis of RCTs to assess the evidence for the efficacy and safety of KTBAMs for KOA in comparison with NSAIDs.

In the previous systematic reviews of the efficacy and safety of traditional Chinese medicine prescriptions in the treatment of KOA, the included studies compared different prescriptions with different efficacies and mechanisms of action, and there was a high level of clinical heterogeneity among the studies [23, 24]. In contrast, in the present study, the interventions were strictly limited to KTBAMs, and the control measures were limited to NSAIDs. To some extent, the bias caused by heterogeneous sources and drugs with different mechanisms of action was reduced, and the results of this study have higher clinical significance. In addition, our study incorporated additional and updated clinical research reports, which complemented and updated the previous systematic reviews. In conclusion, it is necessary to study the efficacy and safety of KTBAMs in the treatment of KOA.

## 2. Materials and Methods

### 2.1. Literature Search

Seven databases, including PubMed, EMBASE, Cochrane Central Register of Controlled Trials, China National Knowledge Infrastructure, Chinese Scientific Journal Database, Wanfang Data and Chinese Biomedical Literature Database, were investigated from their inception through August 2019. The reference lists of retrieved papers were also studied. The following search terms were used individually or in combination. The mesh terms in this paper are as follows: “osteoarthritis, knee,” “Anti-Inflammatory Agents, Non-Steroidal,” and the entry terms are as follows: “Bushen,” “Kidney-tonifying,” “Blood-activating,” “arthritis,” “osteoarthritis,” “knee osteoarthritis,” “knee arthritis,” and “osteoarthritis of knee joint.” To increase the search range, no date and no language limits were imposed. Additionally, no restrictions on population characteristics were imposed. The specific search strategies for PubMed and EMBASE searches are shown in the Supplemental Table.

### 2.2. Study Selection

Two reviewers (HTH and JKP) screened the abstracts of all retrieved titles and decided whether the paper contained potentially relevant data. Disagreements were discussed with another author (JL). The eligibility criteria for this meta-analysis were RCTs comparing KTBAMs with NSAIDs for the treatment of KOA.

The inclusion criteria were as follows: (a) patients were diagnosed with KOA. The diagnosis of participants was in accordance with the recognized criteria for KOA, such as the guidelines established by the American College of Rheumatology in 1995. (b) The treatment group was treated with a KTBAM (the traditional Chinese medicine prescription must have contained both a recognized CHM with kidney-tonifying effects and a CHM with blood-activating effects). The control group was treated with NSAIDs. In addition, the treatment duration was required to be at least 2 weeks, more than 10 subjects were assessed in each group, and the original data were available. (c) The types of study included RCTs that were not limited based on concealment, blinding methods, and allocation schemes, and the language was limited to Chinese and English. The sex, age, and source of the subjects were not limited.

The exclusion criteria were as follows: (a) multiple publications reporting on the same groups of participants were excluded to reduce overlapping data; (b) participants were excluded if they had rheumatoid arthritis, ankylosing arthritis, joint tuberculosis, purulent arthritis, allergic arthritis, Kashin–Beck disease, or podagra; (c) case reports, letters, editorials, and nonhuman studies were excluded; and (d) studies from which the relevant data could not be extracted were excluded.

### 2.3. Data Extraction

Data extraction included the first author's name, year of publication, sample size, diagnostic criteria, age and sex of the participants, details of the intervention and control conditions, treatment duration, and outcome measurements for each study. Two authors (HTH and WYY) independently conducted the data extraction according to predefined criteria. Any uncertainty was resolved through discussion with another author (JL). The reasons for exclusion were recorded. The data were extracted from the included RCTs to a predefined Excel table (Microsoft Corp, Redmond, WA) and cross-checked by the two reviewers (HTH and WYY)

### 2.4. Risk of Bias and Quality Assessment

Two authors (HTH and HDL) independently assessed the methodological quality of each trial according to the standards advised by the Cochrane Handbook [25]. Disagreements, if any, were resolved by discussion and reached consensus through a third reviewer (JKP). The risk of bias was evaluated for each study by assessing the randomization process, the treatment allocation concealment, the blinding of participants and personnel, the blinding of outcome assessment, the completeness of the data, the reporting of results, and other biases. Selective reporting bias was judged according to the published protocols for the registered clinical trials that were contained on the Chinese clinical trial registry (http://www.chictr.org) and international clinical trial registry of the US National Institutes of Health (http://clinicaltrials.gov) websites. We compared the outcome measures between the study protocol and the final published trial.

### 2.5. Data Analysis

Data analysis was carried out using Review Manager software (V.5.3) provided by the Cochrane Collaboration. Given the characteristics of the extracted data in the review, continuous outcomes were expressed as the mean differences (MDs) with 95% confidence intervals (CIs). Differences in categorical variables were expressed as risk ratio (RR) values and 95% CIs. Heterogeneity was assessed by means of *I*^2^ statistics. *I*^2^ ≥ 50% represented high heterogeneity. A standardized mean difference (SMD) was used when the studies included in the meta-analysis assessed the outcome based on different scales (e.g., visual analog scale (VAS) 0-10 and VAS 0-100). Initially, a fixed-effect model would be used to compare the outcomes, unless the heterogeneity tests indicated that the *I*^2^ statistic ≥50% and substantial heterogeneity existed between studies; in this case, the reasons for this heterogeneity would be searched for and a random-effect model would be used for comparison. The subgroup analysis was undertaken according to prespecified criteria to investigate heterogeneous results or to determine the effect of prespecified criteria on the pooled estimate. We assumed that clinical differences would mainly originate from the treatment duration and the dosage and frequency of NSAIDs; therefore, subgroups were divided based on these factors. Publication bias was analyzed by funnel plot analysis if sufficient studies (*n* ≥ 10) were found.

### 2.6. GRADE the Evidence

The GRADE system was used to evaluate the quality of the evidence for each outcome [25]. GRADE-pro GDT Online Tools (available on https://gradepro.org/) were used to evaluate the evidence regarding the included outcomes. Initially, RCTs were considered to be of high confidence in estimating an effect, and observational studies were considered to be of low confidence in estimating an effect. The reasons that may decrease the level of confidence included risk of bias, inconsistency, indirectness, imprecision, and publication bias. The reasons that may increase the level of confidence included a large effect, dose response, and accounting for all plausible residual confounding and bias. The GRADE evidence was divided into the following categories: (1) high-quality evidence, which indicated that further research was unlikely to change the confidence in the estimate of the effect; (2) moderate-quality evidence, which indicated that further research was likely to have an important impact on the confidence in the estimate of the effect and may change the estimate; (3) low-quality evidence, which indicated that further research was likely to have an important impact on confidence in the estimate of the effect and was likely to change the estimate; and (4) very low-quality evidence, which indicated that we were very uncertain about the results.

## 3. Results

### 3.1. Description of Included Studies

**Figure 1:**
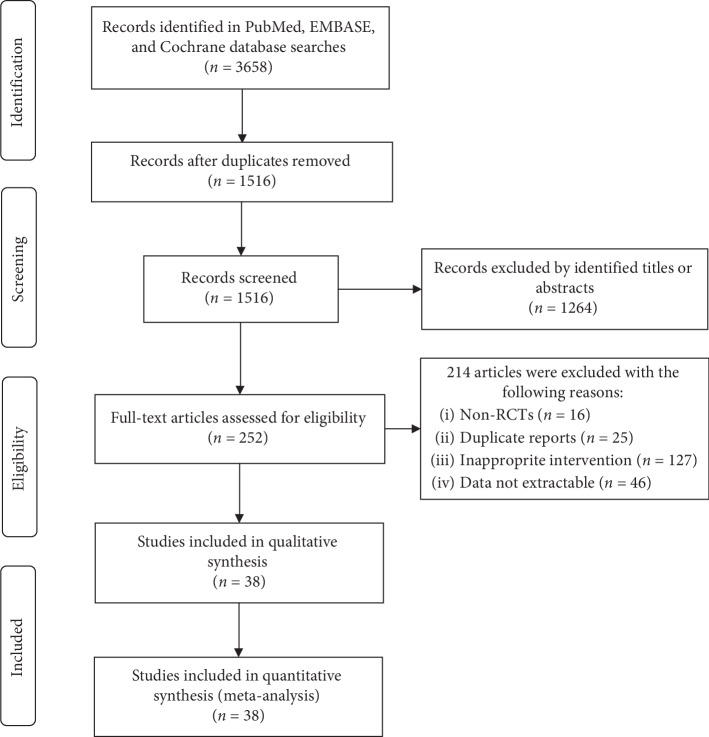
Flow chart showing study identification, review, and selection.

The results of searching strategies are shown in Figure 1. In total, 3685 records were obtained through database searches, and 252 potentially relevant articles were identified after screening the titles and abstracts. According to the selection criteria, 214 articles were excluded. Finally, 38 RCTs [19, 26–62] were included in the review. One study was conducted in Thailand [19], and the other studies were conducted in China. The language of the enrolled trials included English and Chinese.

The essential characteristics of the 38 studies [19, 26–62] are described in Table 1. All the studies, including 2012 patients from the treatment group and 1982 individuals in the control group, were recruited into this systematic review. Two different diagnostic criteria of KOA were used in most of the included trials: 19 studies [19, 30, 36–38, 41, 42, 44–54, 58] used the 1995 American College of Rheumatology Guidelines for The Medical Management of Osteoarthritis (ACR criteria-1995) and 15 studies [26, 27, 31, 33–35, 39, 40, 43, 55–57, 60–62] used the 2007 Chinese Medical Association Guidelines for The Diagnosis and Treatment of Osteoarthritis (CMA criteria-2007). One study [28] used the Guiding Principles for Clinical Research of New Chinese Medicine, two studies [29, 32] referred to the Criteria for the Diagnosis and Therapeutic Effect of Diseases and Syndromes in Traditional Chinese Medicine, and only one study [59] did not explicitly mention diagnostic criteria. The two sets of criteria for KOA in China were basically the same and consistent with the criteria in America and depend mostly on a diagnosis of clinical manifestations and a knee joint X-ray. The patients enrolled in the review ranged from 48 to 65 years of age.

In total, 3994 participants were involved in the 38 RCTs; nine studies [29, 37, 39, 41, 45, 52, 53, 58, 59] did not provide information on the participants' sex; the remaining 29 RCTs [19, 26–28, 30–36, 40, 42–44, 46–51, 54–57, 60–62] included 1192 male participants and 2006 female participants. The RCTs recruited people with durations of treatment that varied from 2 weeks to 12 weeks. Only five studies [19, 33, 39, 49, 60] reported baseline severity of KOA with a Kellgren–Lawrence X-ray of the participants. The other studies did not provide information on baseline disease severity, but all reported baseline balance measures.

All studies investigated oral KTBAMs, including multi-ingredient CHM, and the top 20 forms used based on frequency are listed (Table 2). The preparation forms of the multi-ingredient CHM formulas were decoctions in 28 studies [28–30, 32, 35–39, 41–50, 52–55, 57–60, 62], tablets in two studies [40, 51], capsules in five studies [27, 33, 34, 56, 61], and pills in the others [19, 26, 31]. All studies used KTBAMs compared with NSAIDs. Among the included trials, KTBAMS were compared with meloxicam in six studies [30, 32, 35, 41, 47, 62], with celecoxib in 17 studies [26, 31, 34, 36, 38–40, 42, 43, 45, 48, 49, 56, 57, 59–61], with diacerein in three studies [28, 33, 58], with diclofenac in six studies [19, 27, 46, 52–54], with loxoprofen in two studies [37, 55], with fenbid in two studies [44, 50], and with ibuprofen [51] or etodolac [29] in the remaining studies.

All studies provided treatment of equal duration in the intervention and control groups. The treatment durations were 2 weeks, 4 weeks, 5 weeks, 6 weeks, 8 weeks, and 12 weeks. Only one study [31] had a posttreatment follow-up phase of 4 weeks. Ten studies [19, 31, 38–40, 48, 56, 58, 60, 62] reported dropouts during the treatment phase with reasons provided. None of the dropouts were due to serious adverse events (AEs). Studies were considered to have no dropouts when they reported equal numbers.

### 3.2. Quality Assessment

**Figure 2:**
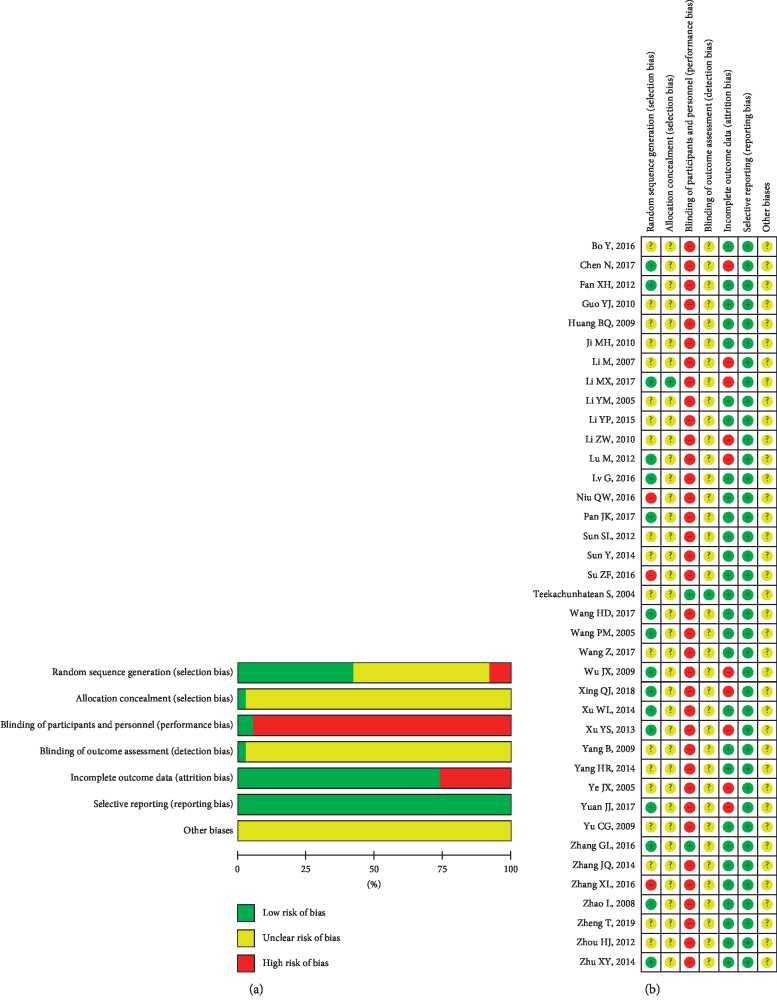
Risk of bias assessment. Note: (a) Risk of bias graph: review authors' judgments about each risk of bias item presented as percentages across all included studies. (b). Risk of bias summary: review authors' judgments about each risk of bias item for each included study (“+” indicates a low risk of bias, “−” indicates a high risk of bias, and “?” indicates an unclear or unknown risk of bias).

**Figure 3:**
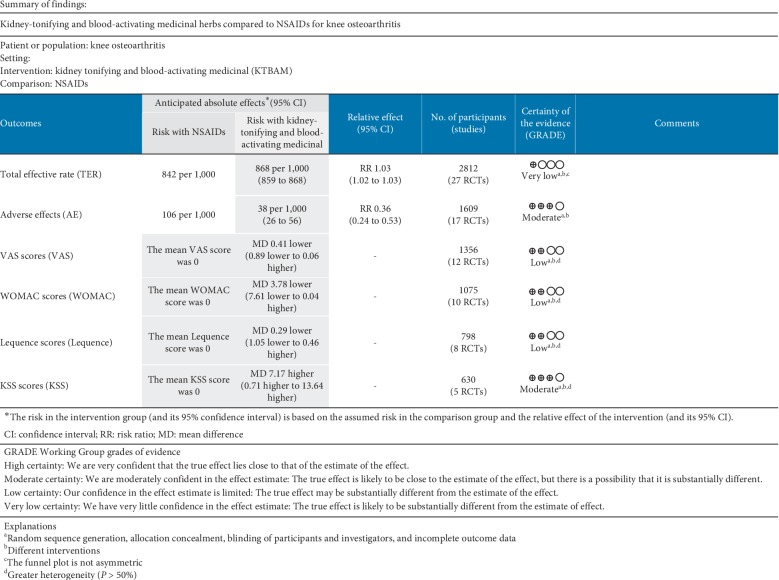
Grade of evidence evaluation based on GRADE Working Group.

Sixteen studies [28, 29, 33, 34,39, 40, 42, 48, 49, 53, 55, 56, 58, 60–62] used a random number table and were evaluated as low risk. The others [19, 30, 32, 35–38, 41, 43–47, 50–52, 54, 57, 59] had unclear risk for not providing detailed information regarding random sequence generation. Three studies [26, 27, 31] were assessed as high risk because they generated the sequence according to the order in which the patients attended the clinic. For allocation concealment, one study [58] was at low risk for using allocation concealment, and other studies were at unclear risk because they lacked the relevant information. Two studies [19, 29] were assessed as having low risk for blinding of participants and personnel because placebo control groups were established. Other studies [26–28, 30–62] were assessed as having a high risk for blinding participants and personnel because of the absence of placebo controls. One study [19] was at low risk for the blinding of outcome assessors; other studies [26–62] were at unclear risk because they provided no such information. Ten studies [19, 31, 38–40, 48, 56, 58, 60, 62] were at high risk for incomplete outcome data because they did not include dropouts in their posttreatment data analysis. The other studies [26–30, 32–37, 41–47, 49–55, 57, 59, 61] were at low risk. All studies had a low risk for selective outcome reporting because their methods matched the results and had an unclear risk for other biases (Figure 2). The results of the GRADE analysis are presented in Figure 3.

### 3.3. Outcomes

The primary outcomes of this meta-analysis were “total effective rate” and “adverse effects,” and the secondary outcomes were “VAS scores,” “WOMAC scores,” “Lequence scores,” and “KSS scores.”

#### 3.3.1. Total Effective Rate

The evaluation of the total effective rate was based on the Guiding Principles for Clinical Research of New Chinese Medicine formulated by the State Food and Drug Administration of China [63]. The effective rate was graded into 4 categories: (1) Clinically controlled: pain and other symptoms disappeared, joint activity was normal, integral decreased ≥95%, and X-ray was normal. (2) Significantly improved: pain and other symptoms disappeared, joint movement was not limited, integral decreased ≥70% and <95%, and X-ray showed a marked improvement. (3) Improved: pain and other symptoms were basically eliminated, joint movement was slightly limited, integral decreased ≥30% and <70%, and X-ray showed improvement. (4) Ineffective: symptoms such as pain and joint activity did not improve significantly, integral decreased <30%, and X-ray did not change. The effective rate was calculated as follows: (total pretreatment score − total posttreatment score)/total pretreatment score × 100%. The total effective rate was calculated as follows: (number of clinically controlled + number of significantly improved + number of improved)/total number of cases × 100%.

**Figure 4:**
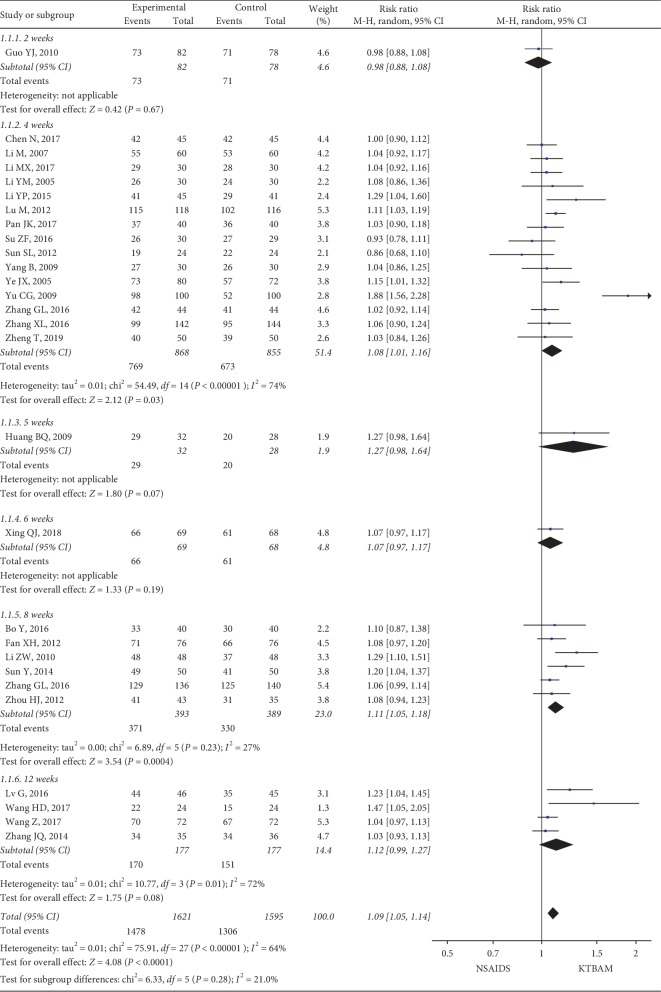
Forest plot of the effect of KTBAMs versus NSAIDs on the total effective rate.

The review found that the total effective rates were reported in 27 studies [26, 28–32, 35, 36, 38, 40, 42, 45, 46, 50–52, 55–59, 61, 62], and the post-follow-up effectiveness in these 27 studies and the total sample were analyzed in a random-effect model (Figure 4). KTBAMs were significantly more effective than NSAIDs (*n* = 2784, RR = 1.09, 95% CI 1.05 to 1.14, *P* < 0.00001; *I*^2^ = 64%) at the end of the treatment phase. Considering that 2 weeks, 4 weeks, 5 weeks, 6 weeks, 8 weeks, and 12 weeks were commonly used treatment durations, a subgroup meta-analysis according to the treatment duration was conducted. The effectiveness of oral KTBAMs in the studies with 4 weeks of treatment (RR = 1.08, 95% CI 1.01 to 1.16, *P*=0.03) was consistent with that of the studies with 8 weeks of treatment (RR = 1.11, 95% CI 1.05 to 1.18, *P*=0.0004), as well as the overall effect of all studies.

#### 3.3.2. Adverse Effects

**Figure 5:**
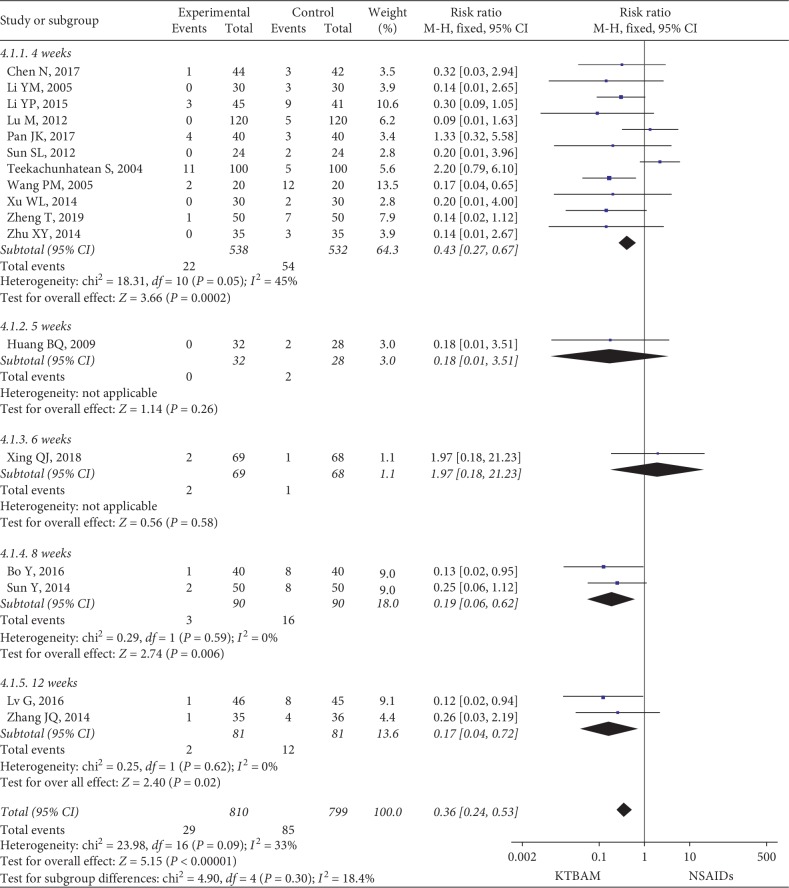
Forest plot of the safety of KTBAMs versus NSAIDs based on adverse events (AEs).

A total of 17 studies [19, 28, 30, 32–36, 40, 41, 47, 52, 53, 56, 57, 61, 62] reported adverse effects (Figure 5) but these were not mentioned in the other studies. Commonly seen AEs were gastrointestinal symptoms (including nausea/vomiting, dyspepsia, diarrhea, and constipation). Increased blood pressure and central nervous system symptoms were also reported. No abnormalities were reported in the routine blood examinations or in liver and renal function. However, adverse drug reactions were not reported, and none of the adverse effects were serious in either group.

All studies were pooled into meta-analyses. KTBAMs demonstrated a lower rate of occurrence of AEs (RR = 0.36, 95% CI 0.24 to 0.53, *P* < 0.00001, with low heterogeneity, *I*^2^ = 33%). Similarly, subgroup analyses of 4 weeks (RR = 0.43, 95% CI 0.27 to 0.67, *P*=0.0002), 8 weeks (RR = 0.19, 95% CI 0.06 to 0.62, *P*=0.006), and 12 weeks (RR = 0.17, 95% CI 0.04 to 0.72, *P*=0.02) were consistent with the pooled effect. The results at 5 weeks (RR = 0.18, 95% CI 0.01 to 3.51) and 6 weeks (RR = 1.97, 95% CI 0.18 to 21.23) indicated no significant differences between the groups.

#### 3.3.3. VAS Scores

**Figure 6:**
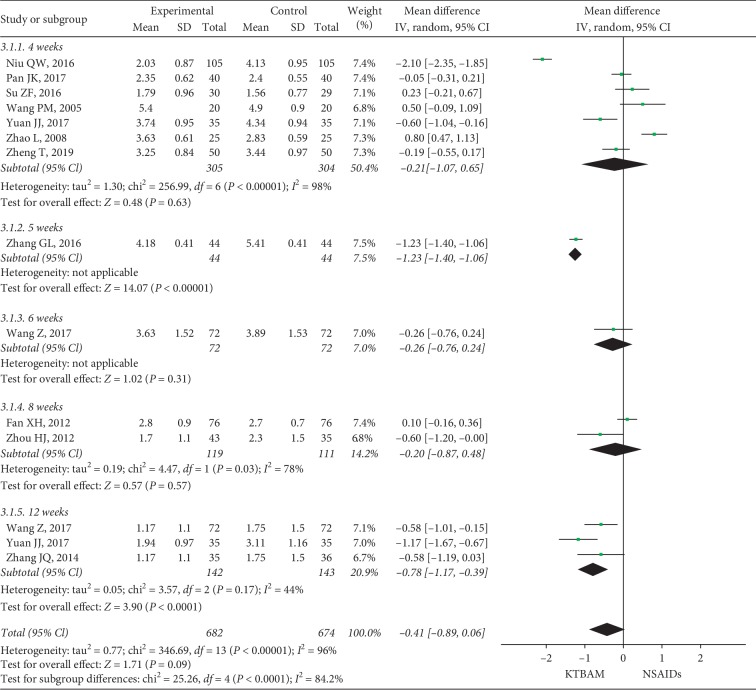
Forest plot of the effect of KTBAMs versus NSAIDs on VAS scores.

For the VAS scores, twelve studies [26, 27, 29, 36, 38, 42, 49, 53, 57, 59–61] evaluated the effects of KTBAMs compared with conventional NSAIDs (Figure 6). Data from one study [19] were excluded because the assessments of the VAS scores included pain and stiffness, which was different from the other studies. The mean differences in VAS scores were not significantly different between the KTBAM group and the NSAID group after 4 weeks (MD = 0.21, 95% CI −1.07 to 0.56, *P*=0.63), 6 weeks (MD = −0.26, 95% CI −0.76 to 0.24, *P*=0.31), and 8 weeks (MD = −0.20, 95% CI −0.87 to 0.21, *P*=0.48), as well as for the overall effect across all studies (MD = −0.41, 95% CI −0.89 to 0.06, *P* < 0.09, with high heterogeneity, *I*^2^ = 96). However, the KTBAM group seemed to have an advantage over the NSAID group in the comparison of VAS scores after 5 weeks (MD = −1.23, 95% CI −1.40 to −1.06, *P* < 0.00001) and 12 weeks (MD = −0.78, 95% CI −1.17 to −0.39, *P* < 0.0001) of treatment.

#### 3.3.4. WOMAC Scores

**Figure 7:**
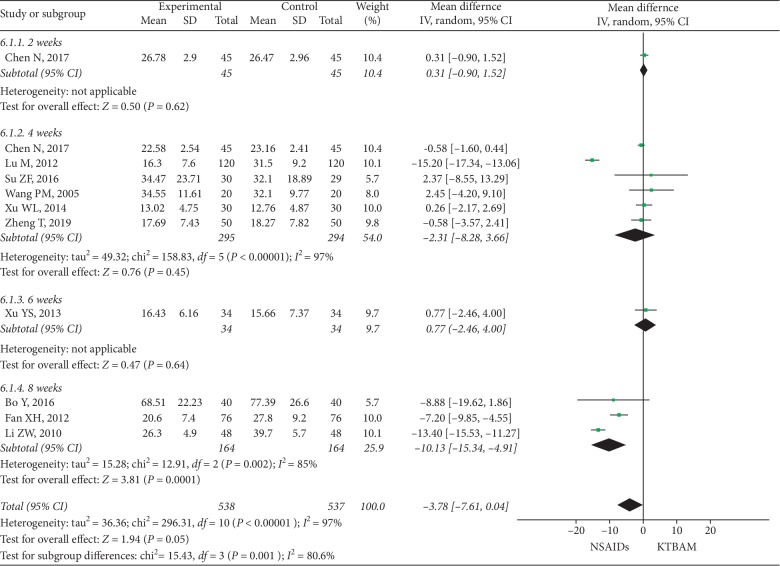
Forest plot of the effect of KTBAMs versus NSAIDs on WOMAC scores.

Ten studies [26, 30, 34, 39, 40, 42, 53, 54, 56, 57] measured WOMAC scores to evaluate the efficacy of treatment (Figure 7). Of these, all ten studies were pooled and analyzed, with 538 participants in the KTBAM group and 537 in the NSAID group. The aggregated result in the random-effect model showed a significant difference (MD = −3.78, 95% CI −7.61 to 0.04, *P*=0.05) between KTBAMs and NSAIDs on WOMAC scores with heterogeneity (*I*^2^ = 97%). The subgroup analyses based on treatment durations of 2 weeks, 4 weeks, and 6 weeks were consistent with the pooled result (2 weeks: MD = 0.31, 95% CI −0.90 to 1.52, *P*=0.62; 4 weeks: MD = −2.31, 95% CI −8.28 to 3.66, *P*=0.45; 6 weeks: MD = 0.77, 95% CI −2.46 to 4.00, *P*=0.64). For comparison, the 8-week duration of treatment included only two studies [42, 54] and showed a favor toward NSAIDs (MD = −10.13, 95% CI −15.34 to −4.91, *P*=0.0001). Two studies used diclofenac [53, 54] as a comparison, one study used meloxicam [30], and the remaining used celecoxib [26, 34, 39, 40, 42, 56, 57]. In the subgroup analysis based on type of NSAID, the meta-analysis results for these groups were consistent with the overall meta-analysis results and suggested a high level of heterogeneity.

#### 3.3.5. Lequence Scores

**Figure 8:**
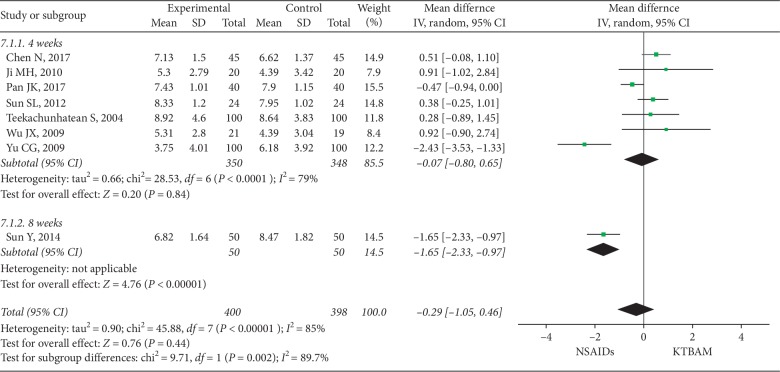
Forest plot of the effect of KTBAMs versus NSAIDs on Lequence scores.

Lequence scores were reported by eight studies [19, 35, 41, 43, 44, 48, 56, 61] (Figure 8) as an outcome to assess the efficacy of treatment. The pooled results of the meta-analysis suggested no preference for KTBAMs or NSAIDs (*n* = 798; MD = −0.29, 95% CI −1.05 to 0.46, *P*=0.44, *I*^2^ = 85%). A subgroup analysis was performed based on treatment duration, and the result for this group (4 weeks) (7 studies; *n* = 698, MD = −0.07, 95% CI −0.80 to 0.65, *I*^2^ = 79%, *P*=0.84) was consistent with the pooled effect. For the eight-week group, NSAIDs were more effective than KTBAMs (1 study; *n* = 100, MD = −1.65, 95% CI −2.33 to −0.97, *P* < 0.00001).

#### 3.3.6. KSS Scores

**Figure 9:**
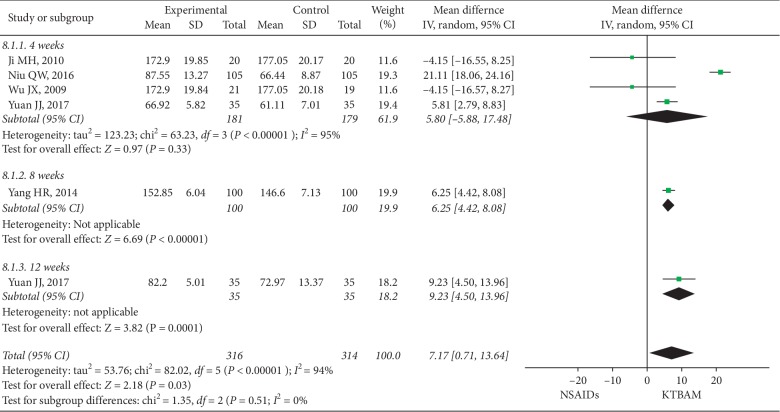
Forest plot of the effect of KTBAMs versus NSAIDs on KSS scores.

Five studies [27, 37, 43, 48, 60] reported Knee Society Scale (KSS) scores (Figure 9), with 316 participants in the KTBAM group and 314 participants in the NSAID group. The mean difference in KSS scores showed a significant difference between the KTBAM group and the NSAID group (MD = 7.17, 95% CI 0.71 to 13.64, *P*=0.03) with high heterogeneity (*I*^2^ = 94%), consistent with the subgroup analysis of 8 weeks and 12 weeks. However, the KTBAM group showed no significant difference in improving KSS scores after 4 weeks (MD = 5.80, 95% CI −5.88 to 17.48, *P*=0.33). Considering the variety of NSAIDs and the similar mechanisms of action of the different drugs, no subgroup of NSAID type could be conducted.

### 3.4. Publication Bias

**Figure 10:**
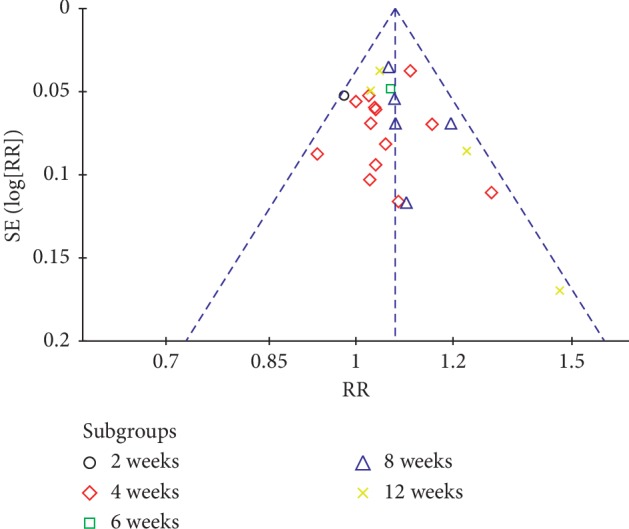
Funnel plot of the total effective rate comparison between KTBAMs and NSAIDs.

Funnel plots were performed for the comparison of KTBAMs and NSAIDs in total effective rate. The results suggested that there was potential bias in the analysis, and either a publication bias or a low-quality small-sample test may have been the main reasons (Figure 10).

## 4. Discussion

Traditional Chinese medicine has been widely used in clinical practice in China as an alternative approach for KOA. Previous studies have demonstrated the efficacy of herbal medicines, such as Duhuo Jisheng decoction (DJD) [64, 65]. Based on traditional Chinese medicine theory, tonifying kidney and activating blood is one of the most common approaches for KOA. DJD is a formula for the treatment of arthralgia and functional disorders by tonifying kidney and eliminating dampness. Our study compared the efficacy and safety of KTBAMs and NSAIDs for KOA from 38 RCTs.

A previous study of a related topic had serious methodological flaws (such as lacking a protocol and containing a noncomprehensive literature search) that existed in another previously published review [66], but the findings were consistent with the results of our study. However, we included 16 additional studies, including one conducted in Thailand, compared with the reviews that included only studies in mainland China. We did not evaluate the clinically controlled rate in our research because its definitions varied across studies making it difficult to obtain an objective result. Additionally, we assumed that the dosage and frequency of oral NSAIDs play a significant role in functional improvement and pain control, although no significant differences were found based on limited data. Overall, we provided a more reliable result in this review.

The meta-analysis indicated that KTBAMs were effective for KOA as follows: (1) 4 weeks of treatment and 8 weeks of treatment with KTBAMs had a higher total effective rate than NSAIDs with the very low-quality evidence; (2) the moderate-quality evidence showed that the adverse reactions from the KTBAMs were lower and the KSS scores were higher; and (3) KTBAMs were neither superior nor inferior to NSAIDs with regards to the VAS scores, WOMAC scores, and Lequence scores based on low-quality evidence. The results of the meta-analysis were inconsistent. Previous research with DJD showed that it can be mainly used to treat arthralgia syndrome, with the effects of eliminating stagnation, removing blood stasis, nourishing the liver and kidney, and invigorating the Qi and blood [19]. As one of the decoctions of KTBAMs, DJD can improve physical function and decrease the pain associated with KOA when combined with conventional Western medicine or other therapies [67]. One reason may be that KTBAMs could produce add-on effects when combined with other therapies. Another reason was the complex combinations of herbs and variations in the dosage forms. Lacking detailed information about the severity of KOA in most studies could have affected the results. The long-term effects of KTBAMs could not be evaluated because of the lack of rigorous reporting of follow-up assessments. Different dosages of Chinese medicine and varied components of the prescriptions, including the use of other types of herbs, may have been a potential bias that influenced our results.

In our research, changes in some measures between the two groups were significantly different after 8 weeks of treatment, but no differences were observed before 8 weeks. Therefore, we supposed that the efficacy of NSAIDs was significantly more stable and reliable than that of KTBAMs (with respect to WOMAC scores, Lequence functional index scores, and KSS scores). The reason why NSAIDs were more reliable may be due to the possibility that KTBAMs may exert their effects via several probable mechanisms from the pharmacodynamic point of view [19], and these mechanisms might have influenced drug concentrations and the subsequent drug effects. As an ancient traditional treatment, KTBAM therapy has developed over thousands of years in China. In the earliest published Chinese medical work, “Inner Classic of the Yellow Emperor” (475 B.C.–221 B.C.), KTBAM therapy was frequently reported as having beneficial outcomes. Despite the lack of knowledge about the biological mechanisms by which Chinese herbal therapy works for knee osteoarthritis, the multitarget therapeutic effect of traditional Chinese medicine has been recognized by many researchers. Some animal experimental studies have indicated that Chinese herbs decreased the levels of nitric oxide in the serum, synovium, and joint cartilage in osteoarthritic rabbits [68]. Another study showed that Du-Huo-Ji-Sheng decoction (a KTBAM compound) exerted significant therapeutic effects in osteoarthritic rabbits, probably through inhibiting the expression of VEGF and hypoxia-inducible factor-1*α* [15]. Yaotongning capsules (a KTBAM compound) promoted proliferation and glycosaminoglycan synthesis in IL-1*β*-induced chondrocytes and may have potential activity in treating chondrocyte degeneration caused by osteoarthritis [69, 70]. A study has found that kidney-tonifying and blood-activating Chinese herbs may suppress the expression of interferon regulatory factor 7 (IRF-7) by regulating the TLR4/MyD88 signaling pathway, resulting in less secretion of interleukin 6 (IL-6) and matrix metallopeptidase 13 (MMP-13), which alleviates inflammation and delays cartilage destruction [71]. Another study reported the effects of low, medium, and high doses of Bushen Huoxue recipe on knee arthritis in rats. It had been found that the pathological changes of knee arthritis in the low-, medium-, and high-dosage groups were gradually alleviated, although the detailed mechanism of action was unknown [72].

KTBAMs had fewer AEs than NSAIDs. The most common AEs were gastrointestinal symptoms, which were similar in the two groups and required no medical intervention. NSAIDs are among the most widely prescribed drugs. Debilitating diseases such as rheumatoid arthritis and osteoarthritis are commonly managed by NSAIDs. However, the pharmacological mechanism of NSAIDs is often associated with the presence of gastrointestinal side effects [73, 74]. NSAIDs are recognized as the most common drugs involved in hypersensitivity drug reactions [75–78]. Until recently, antibiotics, particularly betalactams, were considered the most important inducers of this hypersensitivity [79–81], but NSAIDs are now the leading contributor [77]. However, the present study found that KTBAMs had a lower incidence of adverse reactions than NSAIDs. KTBAMs are mostly natural botanical drugs. Compared with some Aristolochiaceae plants, KTBAMs have less toxic side effects. From the point of view of recognizing and treating diseases in traditional Chinese medicine, the Yin and Yang in harmonious balance indicate health, whereas imbalances to either side indicate unhealthiness, which may result in diseases. CHM is a form of natural plant medicine that mainly treats diseases by adjusting the balance of Yin and Yang and has fewer adverse reactions than Western medications [82]. This suggests that KTBAMs are safe for the management of KOA. However, whether other potential AEs would occur with longer follow-ups was not clear.

The strengths of this review are as follows. First, this is the only study that included patients from different countries in the evaluation of the efficacy and safety of a particular type of CHM for KOA based on the theory of traditional Chinese medicine. Second, this study included 38 studies RCTs with 3994 participants, and subgroup analyses were performed for the different treatment durations and types of NSAIDs, therefore obtaining detailed results. Finally, an intention-to-treat approach was adopted in this meta-analysis to address dropouts; this makes the efficacy results more conservative.

Based on our study, KTBAMs may be an effective and safe alternative treatment in people with KOA when compared with NSAIDs. Additionally, we recommend that KTBAMs be used for less than 8 weeks. However, the quality of the trials included in our research was low; therefore, no firm conclusions could be drawn. Furthermore, the varied composition of the KTBAMs in the formula in these trials and whether these differences play an important role in the efficacy of KTBAMs should be considered. Moreover, most studies did not report the severity of KOA. It is difficult to make a definite conclusion regarding on which patients, based on the level of KOA severity, these findings apply.

Future research should consider including a placebo control, using uniform diagnostic criteria, and using outcome measures according to international guidelines. More trials with well-designed and unified outcome measurements following the Cochrane Handbook should be conducted. Baseline disease severity and AEs should be reported in more detail, and follow-up periods should be used to confirm the long-term effects.

## 5. Limitations

Some limitations of our review downgraded the certainty of these results. First, these RCTs were found mostly in the Chinese literature, with the exception of one in Thailand, and the results were based on evidence with a high risk of bias and low quality; in accordance with the GRADE approach, the overall quality of evidence was limited (ranging from “moderate” to “very low”) due to some serious or very serious limitations. Second, although the outcome measures used in most of these studies are consistent with international guidelines, some of the included studies did not formally report these measures, and the outcome measurements were varied, which led to difficulty in the interpretation of the trials' results. Third, results for the efficacy and safety of KTBAMs versus placebo were not available from this review because only one of the included studies used a placebo control; therefore, placebo effects were not completely eliminated. Fourth, the long-term effects of KTBAMs remain uncertain because of the lack of outcome measures used in the follow-up phase. Finally, the heterogeneity across studies was high, mainly owing to the methodological flaws and the use of different medications.

## 6. Conclusion

KTBAMs appear to be as effective as NSAIDs and seem to have an add-on effect to NSAIDs for the treatment of KOA. Additionally, KTBAMs appeared safe for KOA patients because of the lower rate of occurrence of AEs than NSAIDs, but NSAIDs appeared to have a more reliable effect than KTBAMs after 8 weeks of treatment. However, the methodological limitations reduced the confidence in the effect estimates in this research. More high-quality RCTs with unified measurements and guidelines are needed in the future to precisely assess the effectiveness and safety of KTBAMs.

## Figures and Tables

**Table 1:** Basic characteristics of the included studies.

First author, year, country	Sample size (T/C)	Age, mean ± SD (year)	Sex (M/F)	Treatment group	Control group	Treatment session (week)	Outcome assessment
Teekachunhatean S, 2004, Thailand [19]	200 (100/100)	T: 62.66 ± 9.46 C: 62.38 ± 8.22	T: 22/78 C: 19/81	Duhuo jisheng pills	Diclofenac 25 mg/time, PO, TID	4	LES, VS, AE
Su ZF, 2016, China [26]	59 (30/29)	T: 58.9 C: 57.1	T: 8/22 C: 9/20	Kanggu zengsheng pills	Celecoxib, 200 mg/time, PO, QD	4	TER, WS, VS
Niu QW, 2016, China [27]	210 (105/105)	T: 59.88 ± 2.41 C: 58.64 ± 2.32	T: 45/60 C: 44/61	Jingwu gutong capsule	Diclofenac sodium tablet, 100 mg/time, PO, QD	4	KS, VS
Lv G, 2016, China [28]	91 (46/45)	T: 54.39 ± 5.24 C: 55.08 ± 4.5	T: 20/26 C: 21/24	Qubi tang	Diacerein, 50 mg/time, PO, BID	12	TER, AE
Zhang GL, 2016, China [29]	88 (44/44)	NA	NA	Cangxi tongbi tang	Etodolac tablet, 400 mg/time, PO, QD	5	TER, VS
Bo Y, 2016, China [30]	80 (40/40)	T: 51.2 C: 49.8	T: 19/21 C: 17/23	Shugan zishen tang	Meloxicam, 7.5 mg/time, PO, QD	8	TER, CCR, WS, AE
Zhang XL, 2016, China [31]	286 (142/144)	T: 55 ± 7 C: 56 ± 8	T: 31/105 C: 35/105	Zhuanggu guanjie pills + placebo	Celecoxib, 200 mg/time, PO, QD + placebo	4	TER, CCR, WS, AE
Li YP, 2015, China [32]	86 (45/41)	T: 54.5 ± 6.2 C: 53.8 ± 6.3	T: 19/26 C: 12/29	Huoxue tongluo bushen fang	Meloxicam, 7.5 mg/time, PO, QD	4	TER, AE
Zhu XY, 2014, China [33]	70 (35/35)	T: 51.79 ± 7.01 C: 48.62 ± 7.51	T: 12/21 C: 15/19	Long bie capsule	Diacerein, 50 mg/time, PO, QD	4	AE
Xu WL, 2014, China [34]	60 (30/30)	T: 55.6 ± 9.3 C: 52.5 ± 11.4	T: 11/19 C: 9/21	Long bie capsule	Celecoxib, 200 mg/time, PO, QD	4	WS, AE
Sun Y, 2014, China [35]	100 (50/50)	T: 48.25 ± 5.87 C: 49.26 ± 5.15	T: 15/35 C: 16/34	Zeng ye run jie tang	Meloxicam, 7.5 mg/time, PO, QD	8	TER, CCR, LES, AE
Zhang JQ, 2014, China [36]	71 (35/36)	T: 60.45 ± 6.53 C: 62.14 ± 8.14	T: 9/26 C: 7/29	Bushen huoxue fang	Celecoxib, 200 mg/time, PO, QD	12	TER, CCR, LYS, VS, AE
Yang HR, 2014, China [37]	200 (100/100)	T: 65 C: 64	NA	Jia wei yang he tang	Loxoprofen sodium, 60 mg/time, PO, BID	8	KS
Zhou HJ, 2012, China [38]	78 (43/35)	T: 53.61 ± 6.37 C: 54.18 ± 6.13	T: 17/26 C: 14/21	Shufu Jiangu decoction	Celecoxib, 200 mg/time, PO, QD	8	TER, CCR, LYS, VS
Xu YS, 2013, China [39]	68 (34/34)	T: 59.17 ± 12.17 C: 62.72 ± 11.64	NA	Bushen huoxue fang	Celecoxib, 200 mg/time, PO, BID	6	WS
Lu M, 2012, China [40]	240 (120/120)	T: 52.93 ± 14.22 C: 54.01 ± 15.35	T: 55/65 C: 50/70	Tenghuang Jiangu tablet	Celecoxib	4	TER, CCR, WS, AE
Sun SL, 2012, China [41]	48 (24/24)	T: 62.75 C: 65.75	NA	Qi teng tang	Meloxicam, 7.5 mg/time, PO, QD	4	TER, LES, AE
Fan XH, 2012, China [42]	152 (76//76)	T: 50.6 ± 8.2 C: 49.8 ± 7.6	T: 29/47 C: 35/41	Jiawei Danggui Sini Tang	Celecoxib, 200 mg/time, PO, QD	8	TER, CCR, WS, VS
Ji MH, 2010, China [43]	40 (20/20)	T: 50.75 ± 2.12 C: 51.02 ± 1.54	T: 9/11 C: 9/11	Buahen huoxue tongluo fang	Celecoxib	4	LES, KS
Yu CG, 2009, China [44]	200 (100/100)	T: NA; 44/46 C: NA; 52/48	T: 44/46 C: 52/48	Bushen huayu tang	Fenbid capsule 200 mg/time, PO, QD	4	TER, LES
Yang B, 2009, China [45]	60 (30/30)	NA	NA	Bushen huoxue medicinal	Celecoxib, 200 mg/time, PO, QD	4	TER, CCR
Guo YJ, 2010, China [46]	160 (82/78)	T: 63.93 C: 61.83	T: 34/48 C: 32/46	Quyu Tongbi decoction	Diclofenac tablet, 75 mg/time, PO, BID	2	TER
Huang BQ, 2009, China [47]	60 (32/28)	T: 50.2 ± 87 C: 51.1 ± 10.2	T: 6/26 C: 11/17	Bushen zhuanggu fang	Meloxicam, 15 mg/time, PO, QD	5	TER, CCR, AE
Wu JX, 2009, China [48]	40 (21/19)	T: 56.5 C: 57.3	T: 4/17 C: 3/16	Duhuo jisheng decoction	Celecoxib	4	LES, KS
Zhao L, 2008, China [49]	50 (25/25)	T: 63.09 ± 7.65 C: 62.65 ± 10.8	T: 2/20 C: 4/19	Bushen huoxue medicinal	Celecoxib	4	VS
Li M, 2007, China [50]	120 (60/60)	T: 57.6 C: 55.2	T: 28/32 C: 27/33	Gu shu tang	Fenbid capsule, 300 mg/time, PO, BID	4	TER, CCR
Ye JX, 2005, China [51]	152 (80/72)	T: 59.2 ± 17.6 C: 57.5 ± 15.3	T: 35/45 C: 31/41	Guanjietong tablet	Ibuprofen capsule, 300 mg/time, PO, QD	4	TER, CCR
Li YM, 2005, China [52]	60 (30/30)	T: 58.4 C: 57.2	NA	Bushen huoxue jianxi fang	Diclofenac tablet, 50 mg/time, PO, BID	4	TER, AE
Wang PM, 2005, China [53]	40 (20/20)	T: 59.85 ± 10.29 C: 60.1 ± 9.5	NA	Xining fang	Diclofenac tablet, 75 mg/time, PO, QD	4	WS, VS, AE
Li ZW, 2010, China [54]	96 (48/48)	T: 52.4 ± 7.6 C: 53.2 ± 8.5	T: 25/23 C: 22/26	Bushen huayu tang	Diclofenac sodium tablet, 75 mg/time, PO, BID	8	TER, WS
Wang HD, 2017, China [55]	48 (24/24)	T: 56.2 ± 8.6 C: 55.7 ± 8.9	T: 14/10 C: 15/9	Bushen huoxue tang	Loxoprofen sodium, 60 mg/time, PO, TID	12	TER
Chen N, 2017, China [56]	90 (45/45)	T: 45.51 C: 45.19	T: 6/39 C: 6/39	Bushen qiangjin capsule	Celecoxib, 200 mg/time, PO, QD	4	VS, WS, LES, TER, AE
Zheng T, 2019, China [57]	100 (50/50)	T: 63.26 ± 4.72 C: 63.12 ± 4.93	T: 27/23 C: 24/26	Bushen huoxue fang	Celecoxib, 100 mg/time, PO, BID	4	VS, WS, TER, AE
Li MX, 2017, China [58]	60 (30/30)	T: 57.33 ± 1.59 C: 60.07 ± 1.61	NA	Bushen huoxue fang	Diacerein, 50 mg/time, PO, QD	4	TER
Wang Z, 2017, China [59]	144 (72/72)	NA	NA	Bushen huoxue tongluo fang	Celecoxib, 100 mg/time, PO, QD	12	TER, VS, LYS
Yuan JJ, 2017, China [60]	70 (35/35)	T: 48.30 ± 5.60 C: 41.20 ± 4.80	T: 18/17 C: 15/20	Bushen huoxue tang	Celecoxib, 200 mg/time, PO, QD	12	VS, KS
Pan JK, 2017, China [61]	80 (40/40)	T: 64.53 ± 6.47 C: 64.55 ± 5.57	T: 6/34 C: 5/35	Longbie capsule	Celecoxib, 200 mg/time, PO, QD	4	TER, VS, LES, AE
Xing QJ, 2018, China [62]	137 (69/68)	T: 51.0 ± 8.0 C: 53.0 ± 9.0	T: 31/38 C: 32/36	Yiqi huayu bushen fang	Meloxicam, 7.5 mg/time, PO, QD	6	TER, VS, WS, AE

Note: TER: total effective rate; CCR: clinical control rate; WS: WOMAC scale; LES: Lequence score; KS: KSS score; LYS: Lysholm score; VS: VAS scale; AE: gastrointestinal adverse reactions; NA: not available; T/C: treatment group/control group; M/F: male/female; SD: standard deviation.

**Table 2:** Top 20 Chinese herbs and efficacy based on frequency of usage in the 38 study prescriptions.

English name	Latin name	Chinese Pinyin name	Frequency of usage
*Kidney-tonifying herbs*			
Achyranthes Root	*Radix achyranthis bidentatae*	Niuxi	24
Prepared Radix Rehmanniae	*Radix rehmanniae preparata*	Shudihuang	18
Malaytea Scurfpea Fruit	*Fructus psoraleae*	Buguzhi	14
Eucommia bark	*Cortex eucommia*	Duzhong	13
Chinese Taxillus Twig	*Herba taxilli*	Sangjisheng	13
Drynaria Fortunei	*Rhizoma drynariae*	Gusuibu	13
Epimedium herb	*Herba epimedii*	Yinyanghuo	10
Common Macrocarpium Fruit	*Fructus corni*	Shanzhuyu	7
Prepared common Monkshood Daughter Root	*Radix Aconiti Lateralis Preparata*	Fuzi	7
*Blood-activating herbs*			
Achyranthes Root	*Radix achyranthis bidentatae*	Niuxi	24
Chinese Angelica	*Radix angelicae sinensis*	Danggui	20
Suberect Spatholobus Stem	*Caulis spatholobi*	Jixueteng	14
Danshen Root	*Radix salviae miltiorrhizae*	Danshen	11
Szechwan Lovage Rhizome	*Rhizoma chuanxiong*	Chuanxiong	10
*Pain relief*			
Doubleteeth Pubescent Angelica Root	*Radix angelicae pubescentis*	Duhuo	10
Clematis Root	*Radix clematidis*	Weilingxian	10
White Peony Root	*Radix paeoniae alba*	Baishao	9
Common Flowering Quince Fruit	*Fructus chaenomelis*	Mugua	9
*Others*			
Licorice Root	*Radix glycyrrhizae*	Gancao	16
Astragalus	*Radix astragalus*	Huangqi	10
Wolfiporia Extensa	*Poria cocos*	Fuling	7
